# The Replanting of a 1.1 from an Ectopic Position during the Course of Orthodontic Therapy: Follow-Up at 8 Years

**DOI:** 10.1155/2019/3956296

**Published:** 2019-12-06

**Authors:** Michele M. Figliuzzi, Maria Altilia, Simone Altilia, Amerigo Giudice, Leonzio Fortunato

**Affiliations:** ^1^School of Dentistry, Department of Periodontics and Oral Sciences, Magna Græcia University, Catanzaro, Italy; ^2^Dentistry School, Tor Vergata University of Rome, Rome, Italy

## Abstract

The case that is reported here describes the replanting of a 1.1 from an ectopic position during orthodontic therapy. The 9-year-old patient suffered from class 2 type malocclusion with the upper maxilla contracted, right-left posterior cross-bite. The clinical case presented the following details: in the upper incisor group, the 1.1 was overlapping the 1.2 and was distalised and completely vestibularised, whilst in the place of the 1.1, a 1.1 supernumerary persisted in occlusion. Following several medical investigations, such as OPT and, most importantly, TC cone beam investigation, the dangerous position of the dental element became clear. This did not present vestibular cortical bone but only gingival mucosa. Following these investigations, the difficulty in bringing the dental element into its natural position through orthodontic treatment became obvious since the natural position was without sufficient bone support. From this, it became obvious that surgery and replanting of the 1.1 immediately after the extraction of the supernumerary 1.1 was the only choice available.

## 1. Introduction

Ectopia is a dental anomaly caused by an alteration of the eruption process in which the element erupts away from its normal seat, in a vestibular, lingual, or palatal position. Dental anomalies can be of genetic, congenital, or acquired origin. The etiopathogenesis can be found in an anomalous position of the dental germ, in the lack of space in the arch where it should lie, and in physiological limits of the deciduous teeth, a precocious loss of a deciduous tooth with subsequent loss of space for the permanent tooth or a basal-dental disharmony. An important aesthetic deficit occurs when ectopia occurs in the anterior section leading to the necessity of orthodontic treatment. Orthodontic dental movement occurs as a result of the remodelling of the bone alveolar and the modification of the periodontal ligaments. The limits of orthodontics are tied to the individual conditions which may modify the success of the treatment; one of these is found in the lack of an adequate connection between the dental element and the alveolar.

## 2. Replanting

The term replanting refers to the insertion of an extracted dental element into an alveolar with consequent temporary splinting. This procedure can be carried out following a trauma which causes the complete extraction of the element or as an intentional surgical operation. In the latter case, an atraumatic extraction is carried out on a dental element which is immediately repositioned in the previously prepared alveolar. In the year 1990, Andersson and Bodin have published a long-term clinical follow-up study [1]. Another study of hypodontia has been published by Symons et al. in 1993 in anomalies associated with hypodontia [2]. In the years 1994 and 1996, Pitt Ford et al. and Bakland and Andreasen identified other influential factors such as postsurgical complications, endodontic healing, periodontal healing, and radicular reabsorption [3, 4].

Barrett and Kenny demonstrated the possibility of success in long-term natural tooth reimplant [5].

Andreasen et al. have published the study of the effect of treatment factors such as treatment delay, repositioning, splinting type, and period and antibiotics [6].

A minimally invasive surgery was proposed by Figliuzzi et al. for the management of impacted maxillary canines [7].

## 3. The Case

**Figure 1:**
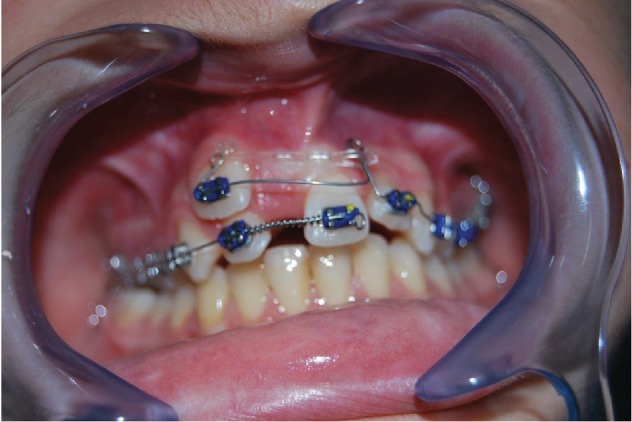
Ectopic permanent 1.1, distalised, vestibularised, and overlapping the 1.2.

**Figure 2:**
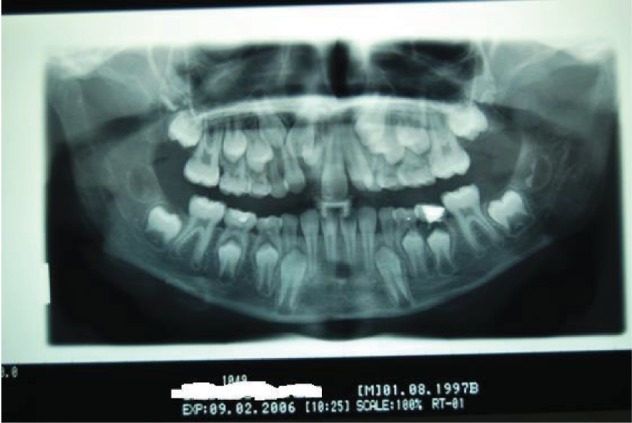
OPT shows the mixed dentition and the presence of the supernumerary 1.1.

**Figure 3:**
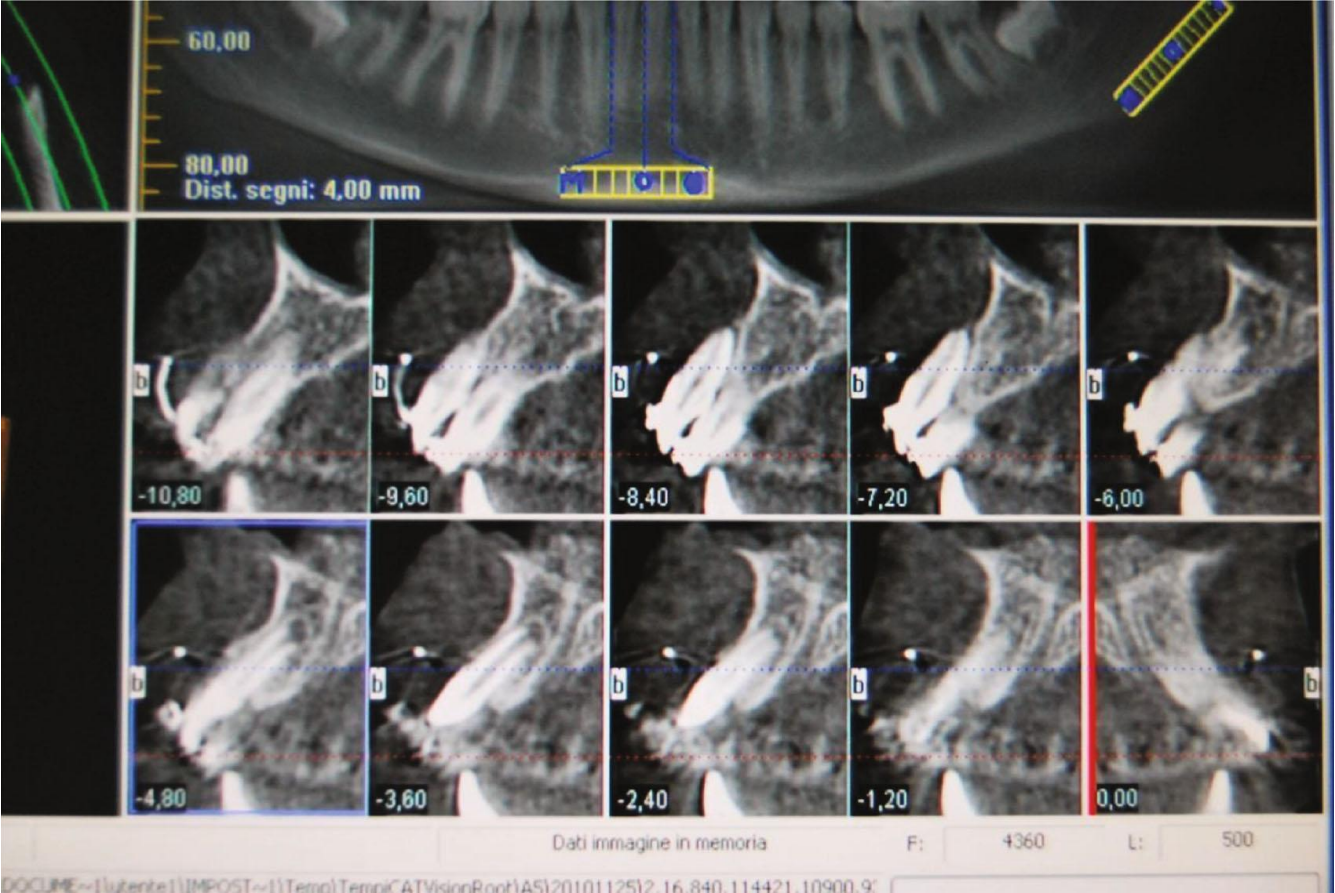
TC cone beam shows the lack of vestibular bone tissue around the permanent 1.1.

**Figure 4:**
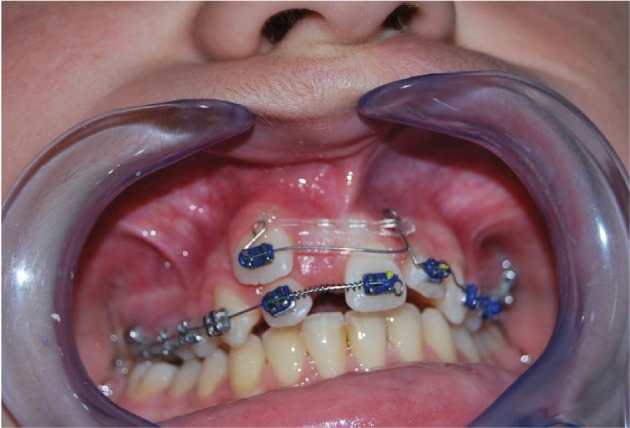
Initial orthodontic treatment.

**Figure 5:**
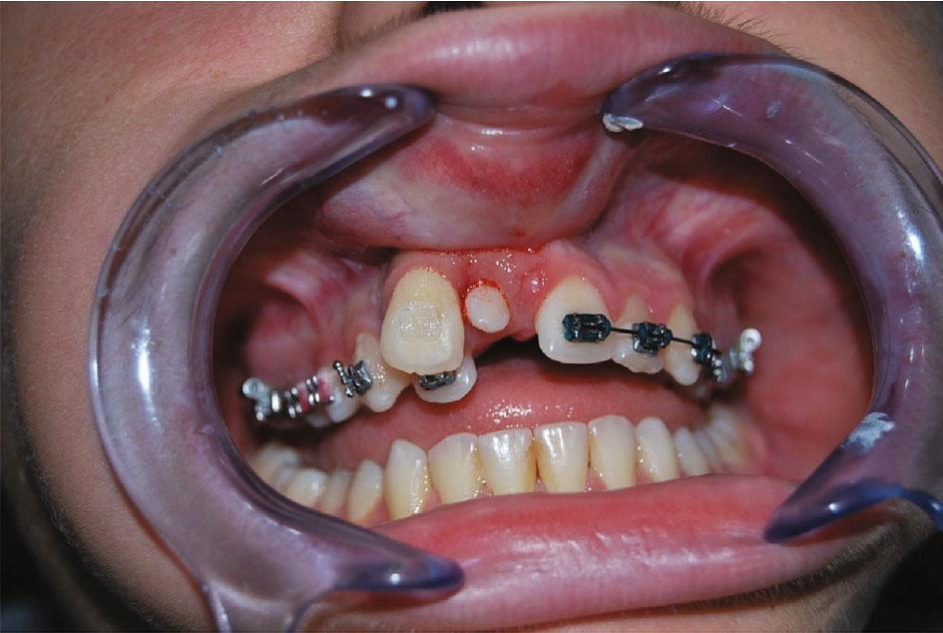
Local anaesthetic.

**Figure 6:**
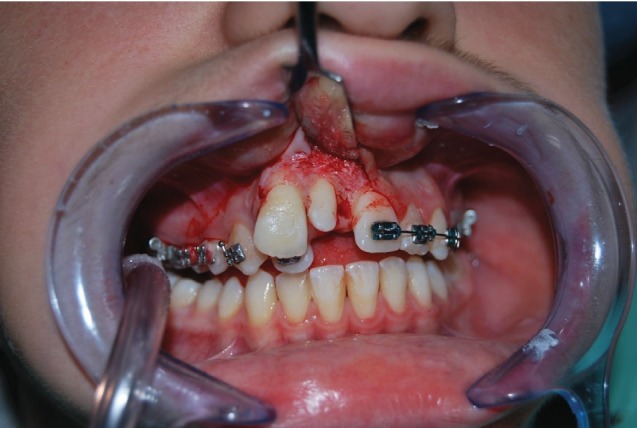
Envelope flap, intrasulcular partial depth from 1.1 to 2.1.

**Figure 7:**
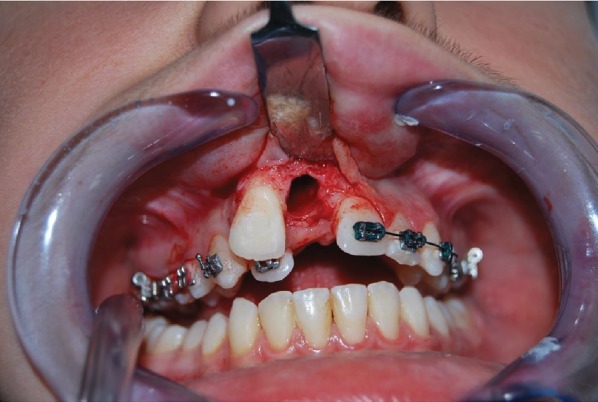
Extraction of the supernumerary 1.1 and preparation of the alveolar.

**Figure 8:**
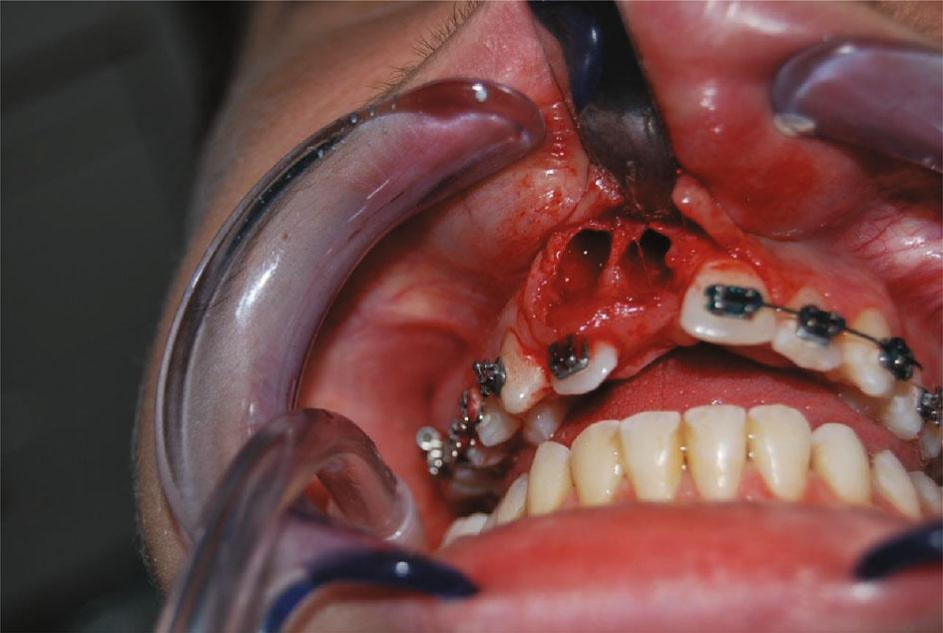
Extraction of the permanent 1.1.

**Figure 9:**
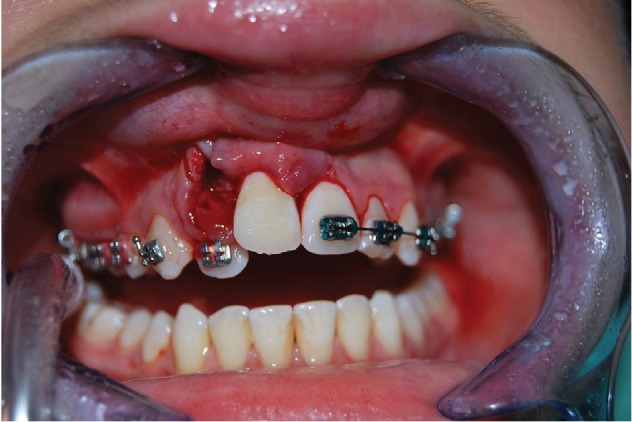
Replanting of the permanent 1.1 in the alveolar of the supernumerary 1.1.

**Figure 10:**
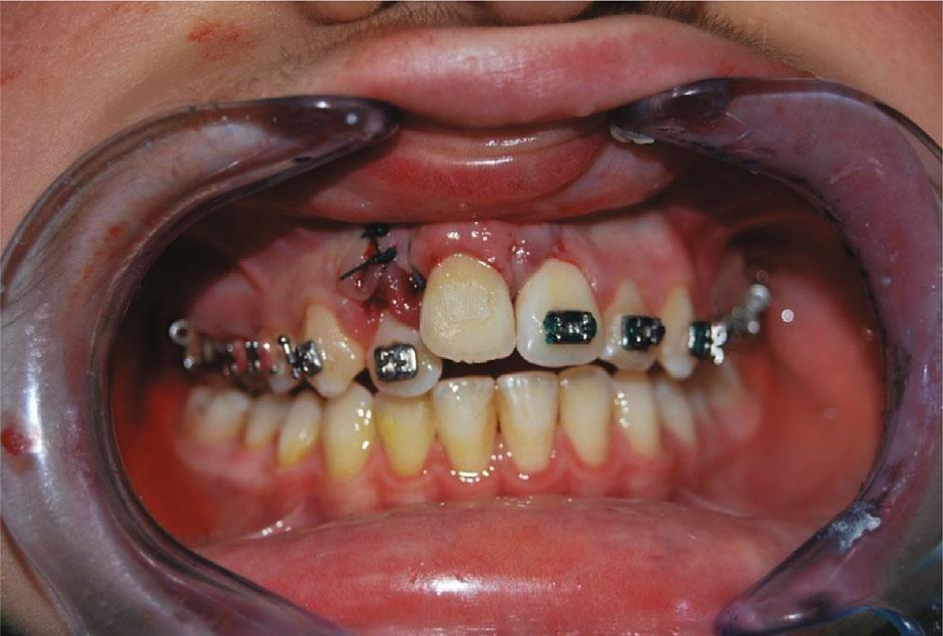
Sutured flap.

**Figure 11:**
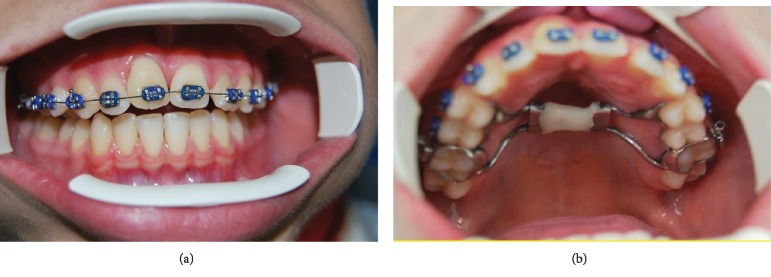
Final orthodontic treatment (a, b).

The 9-year-old male patient (M.G.) presented a class 2 type malocclusion with the upper maxilla contracted and posterior right-left cross-bite as well as an ectopia of the 1.1 that was distalised, vestibularised, and overlapping the 1.2 (Figure 1). The X-rays (Figure 2) showed an intraosseous inclusion of a supernumerary 1.1 in a mixed dentition situation. The presence of the 1.1 supernumerary in bone inclusion determined the ectopia of the permanent 1.1 tooth. Following clinical examination and X-rays, a TC cone beam was requested in order to study the topography of the ectopic element and its relationship with the surrounding structures. The results of the TC cone beam investigation showed the absence of the vestibular bone wall for the dental element (Figure 3) which turned out to be covered only by gingival mucosa. The lack of an adequate relationship between the alveolar and the dental element prevented successful orthodontic therapy; for this reason, the patient was referred for an intentional surgical replanting. The treatment was divided into three phases:

1. *Initial Orthodontic Treatment*. The first phase of the treatment involved orthodontic therapy using a functional arch for 10 to 12 months, a rapid palatal expander, and fixed upper and lower braces (Figure 4).
2. *Immediate Intentional Replanting*. After local infiltration with mepivacaina 1 : 100000 (Figure 5), a vestibular incision was made starting from the central incisor on the right side to the central incisor on the left. The incision, carried out using a Beaver 64, was intrasulcular type, partial depth without vertical release since this system guarantees a better closure of the flap at the end of the operation (Figure 6). After lifting the flap, the supernumerary 1.1 was extracted and the alveolar was suitably prepared to receive the permanent 1.1 (Figure 7). This phase was carried out using a surgical spoon in order to remove any residual bony fragments. At this point, the permanent 1.1 was extracted (Figure 8) and it was immediately replanted in the now empty alveolar which once held the supernumerary 1.1 (Figure 9). The replanted dental element was suitably splinted in order to keep it in place, and the flap was repositioned and sutured after the surgical site was accurately swabbed (Figure 10).
3. *Final Orthodontic Treatment*. Having repositioned the ectopic element in the dental arch, the orthodontic therapy continued with the refining phases to close the case (Figure 11).

**Figure 12:**
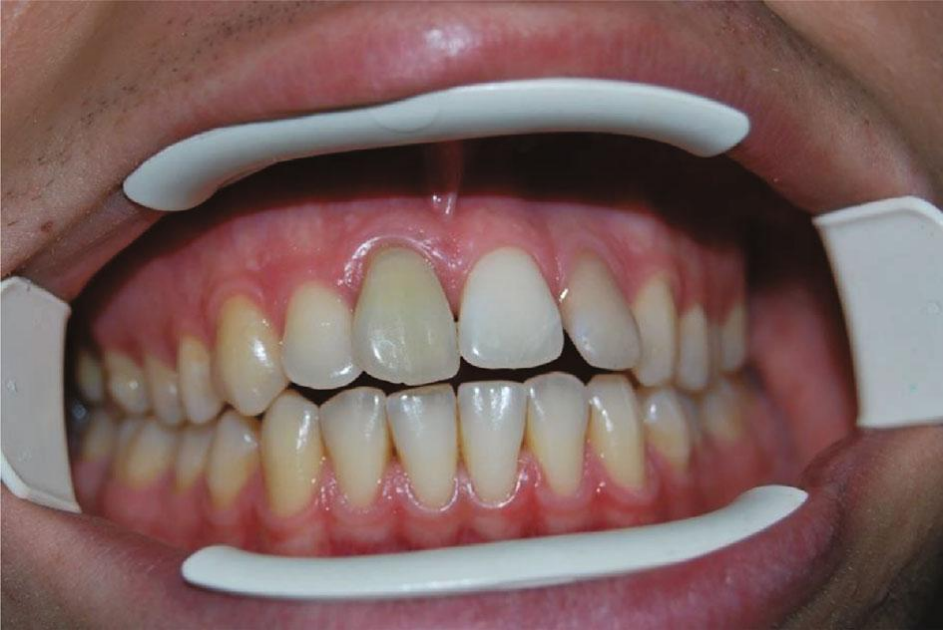
6-year follow-up.

After approximately 1 year, the patient returned for a follow-up checkup and the replanted dental element (1.1) showed signs of necrosis. This was then treated endodontically and followed over a period of time. At the 6-year checkup, the patient was found to have excellent stability of the replanted dental element that had been subsequently treated endodontically (Figure 12).

## 4. Discussion

The biological mechanisms at the basis of intentional replanting are the preservation of the integrity of the periodontal ligament (the main factor in promoting clinical success), atraumatic extraction (fundamental for maintaining the vitality of the element), reduction of the extraoral stay time, and reduction of any osmotic shock of the ligament. Swabbing with water (rather than physiological solution) and lengthened time outside of the alveolar are associated with a greater percentage of radicular reabsorption. Known side-effects of dental replanting are the presence of a complex radicular morphology, lesions to the furcation, periodontal lesions associated with class 3 dental mobility, and radicular caries. The advantages given by the success of replanting are aesthetic and functional recovery, reduced bone loss, absence of postoperative oedema, precise retrograde treatment, short timescale, and reduced risk of complications.
